# Safety and efficacy of low dose pioglitazone compared with standard dose pioglitazone in type 2 diabetes with chronic kidney disease: A randomized controlled trial

**DOI:** 10.1371/journal.pone.0206722

**Published:** 2018-10-31

**Authors:** Bancha Satirapoj, Khanin Watanakijthavonkul, Ouppatham Supasyndh

**Affiliations:** Division of Nephrology, Department of Medicine, Phramongkutklao Hospital and College of Medicine, Bangkok, Thailand; Weill Cornell Medical College Qatar, QATAR

## Abstract

**Background:**

Choices of hypoglycemic agents for patients with type 2 diabetes and chronic kidney disease (CKD) are limited. Available data among patients with CKD suggest that pioglitazone was effective and safe, with no increase in serious adverse effects. However, weight gain and fluid retention are major clinical problems for pioglitazone among patients with CKD. We conducted this study to compare the efficacy and side effects of low dose pioglitazone with standard dose pioglitazone among patients with type 2 diabetes and CKD.

**Methods:**

A total of 75 patients with type 2 diabetes and CKD and inadequate glycemic control receiving any pharmacological antidiabetic treatment were randomly assigned to 2 groups. One group consisted of 37 patients treated with standard dose pioglitazone (15 mg/day) and another group consisted of 38 patients treated with low dose pioglitazone (7.5 mg/day). Glycosylated hemoglobinA1c (HbA1c) and metabolic profiles were monitored every 8 weeks for 24 weeks. Body composition was assessed using bio-electrical impedance analysis (BIA).

**Results:**

After 6 months of therapy, HbA1c levels decreased in both standard and low dose pioglitazone groups. The mean changes in HbA1c for standard and low dose pioglitazone were 1.1±1.6 and -1.4±1.5 (*P* = 0.543), respectively. Compared with low dose pioglitazone, standard dose pioglitazone treatment led to a greater increase in body weight, fat mass, total body water and extracellular water composition. No major adverse effects including hypoglycemia, congestive heart failure and abnormal liver function were identified.

**Conclusion:**

Pioglitazone 7.5 mg once daily treatments presented similar glycemic control to standard dose pioglitazone and exhibited beneficial effects on weight gain and fluid retention among patients with type 2 diabetes and CKD.

## Introduction

Type 2 diabetes mellitus (T2DM) is one of the most important health problems and its disease prevalence has been increasing steadily all over the world. Similarly, the rising incidence of the chronic kidney disease (CKD) will create major problems for both healthcare systems and economies in future years [1]. T2DM is often associated with CKD, and for 30 to 50% of patients receiving dialysis therapy, diabetes is the primary cause of end stage renal disease (ESRD) [2, 3]. Identification and diagnosis of CKD is important to optimize clinical management recommendations for this complex patient population.

Therapeutic options for patients with T2DM and CKD are limited because the reduced glomerular filtration rate (GFR) results in an accumulation of certain drugs potentially leading to adverse side effects [4]. Currently, thiazolidinediones (TZDs), synthetic exogenous agonists of the nuclear peroxisome, proliferator-activated, receptor-gamma (PPARγ), increase insulin sensitivity and inhibit adipose tissue lipolysis as well as possess anti-inflammatory and other beneficial vascular effects [5]. TZDs undergo hepatic metabolism and have been demonstrated to be effective without increasing the risk of hypoglycemic episodes among patients with CKD [6]. The pharmacokinetic profile of TZDS, is similar among subjects with normal or impaired renal function, remaining unaffected even by hemodialysis [7]. Therefore, dosing of TZDs is not a required adjustment among patients with CKD.

Unfortunately, fluid retention and edema have emerged as the most common and serious side effects of TZDs and have become the most frequent cause of discontinued therapy [8, 9]. The mechanism through which TZDs induces fluid retention results from an increase in tubular sodium and water reabsorption in the collecting tubule and an increase in vascular permeability growth factors [10]. Currently, using pioglitazone, 15 to 45 mg daily, is recommended for patients with T2DM. Patients with CKD might constitute a high risk population of TZDs related to fluid retention. Reducing fluid retention with low dose medications is important among those with CKD to continue the beneficial effects to optimize glycemic treatment without affecting the renin-angiotensin-aldosterone system and natriuretic peptides [11, 12]. The aim of the present study was to evaluate the effects of low-dose pioglitazone (7.5 mg/day) on glucose metabolism, and determine side effects related to weight gain and fluid retention and the incidence of edema compared with a standard dose of pioglitazone (15.0 mg/day) among Thai patients with T2DM with CKD.

## Material and methods

### Study design

This research comprised a 24-week randomized, open labeled, controlled study conducted among CKD patients at Phramongkutklao Hospital, Bangkok, Thailand between January 1, 2014, and December 30, 2015. The study was approved by the Ethics Committee of the Institute Review Board at the Royal Thai Army Medical Department December 10, 2013 and all patients gave written informed consent. The study was registered in **Thai Clinical Trials Registry (TCTR20180424002**). The authors confirm that all ongoing and related trials for this drug/intervention are registered. However, our study was registered retrospectively because we were unaware of trial registration and the need to register the clinical trial. Treatment protocols of patients were randomized using a method of block randomization by the researcher to one of two double-blinded treatment groups. A computer-generated randomization procedure in blocks of four was used. The subjects were given pills and compliance was assessed by pill count on follow-up visits.

### Study population

Inclusion criteria of the study included age 18 years or older, diagnosed T2DM with stable glycemic treatment with glycosylated hemoglobinA1c (HbA1c) >8% at least 12 weeks and diagnosed CKD according to Kidney Disease: Improving Global Outcomes (KDIGO) 2012 definition and without history of treatment with TZDs within 12 weeks before starting the study. CKD was defined as having more than three months of GFR<60 mL/min/1.73 m^2^ or evidence of kidney damage including albuminuria and urine sediment abnormalities. Exclusion criteria comprised active malignancy, severe heart, lung or liver disease, stroke, chronic infection, e.g., tuberculosis within one year of starting the study, limited life expectancy within 12 months, edematous state from any cause and specific contra-indications to pioglitazone including increased serum levels of liver enzyme (aspartate aminotransferase [AST] or alanine aminotransferase [ALT] >2.5 times the upper limit of normal) and history of bladder malignancy.

Eligible patients were randomly assigned to two groups. One group consisted of 37 patients treated with standard dose pioglitazone (15 mg/day) orally; the other group consisted of 38 patients treated with low dose pioglitazone (7.5 mg/day) orally. All patients typically continued their normal daily activities during both treatments and were instructed to adhere to a disease and weight-oriented diet and exercise regimen throughout the study. To give 80% power at p < .05 to detect a difference in incidence of peripheral edema from 3.7% to 26.8% a total of 38 patients per group were required, where 76 is the total recruitment. [12]

### Physical, biochemical measurements and body composition

Initial screening included medical history, physical examination and routine blood tests. Every eight weeks, all patients were monitored regarding body weight, body mass index (BMI), systolic blood pressure (SBP), diastolic blood pressure (DBP), symptoms and signs suggestive of adverse effects and compliance with the medication, dietary therapy and exercise therapy. BP was measured with the patient in the sitting position after at least five minutes of rest. All subjects underwent laboratory blood tests every eight weeks during the study period. Serum samples and urine analysis were obtained after overnight fasting and were immediately processed for analysis including fasting plasma glucose (FPG), HbA1C, low density lipoprotein cholesterol (LDL), AST, ALT, urine sodium and urine creatinine.

Direct segmental multifrequency bioelectrical impedance analysis (DSM-BIA) was performed using the In-Body (720) body composition analyzer. This equipment has been shown to have high test-pretest reliability and accuracy. [13] The spectrum of electrical frequencies was used to predict body composition, total fat mass, intracellular water (ICW) and extracellular water (ECW) compartments of the total body water (TBW) in the various body segments. Body composition was assessed at baseline and every eight weeks of the study period.

### Safety monitoring

Adverse events that were or were not considered to be related to pioglitazone treatment were monitored every eight weeks. The patients were questioned each time in a systematic way about their experiences concerning adverse events during the previous eight weeks.

### Statistical analysis

Data were summarized in frequencies (or percentages) for categorical variables and as means ±SD for continuous variables. The chi-square test (or Fisher’s exact) and the two-sample t-test were used to compare differences between the groups for categorical and continuous variables, respectively. Changes in continuous measures between baseline and study periods were tested by means of the paired t-test, and any differences in these changes between groups were tested using repeated ANOVA of variance for repeated measures. Nonparametric methods were used for nonnormally distributed values. All results were considered significant when *P* was <0.05. Statistical analyses were performed using SPSS, Version 15.0.

## Results

**Fig 1:**
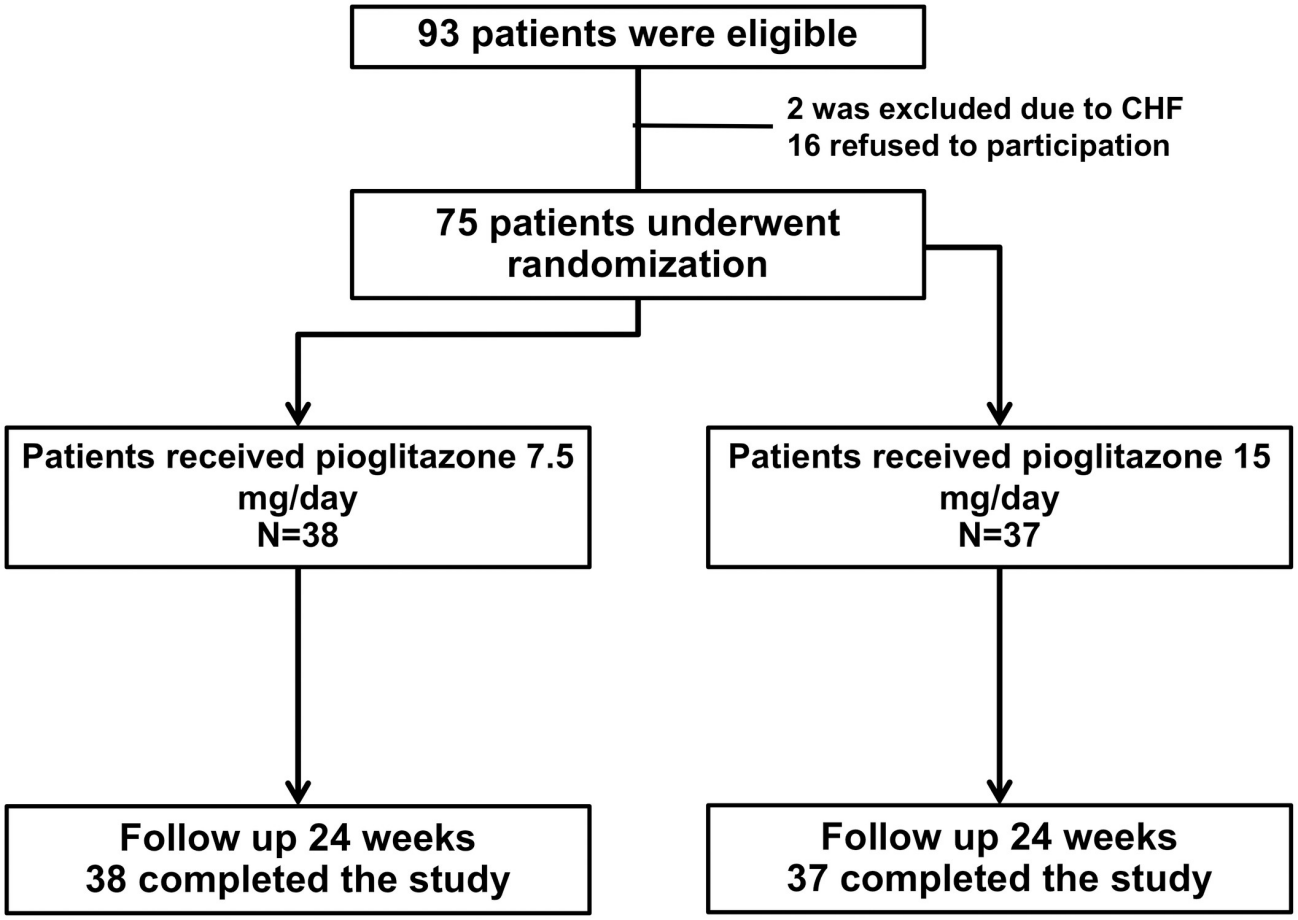
Flow chart study.

A total of 93 patients in the outpatient clinic were screened for possible study enrollment. Seventy-five patients were eligible following the study criteria as shown in Fig 1. Patients with T2DM with mean age 62.2±10.6 years, mean GFR 64.1±30.3 mL/min/1.73 m^2^ and mean BMI 26.6±3.4 kg/m^2^ were eligible according to the entry criteria. The low dose pioglitazone group consisted of 38 patients treated with pioglitazone (7.5 mg/day) orally and the standard dose pioglitazone group consisted of 37 patients treated with pioglitazone (15.0 mg/day) orally. Characteristics of the study population are shown in Table 1. Main CKD stages were stage II (30.7%), stage III (37.3%) and stage IV (25.3%). The baseline characteristics of both groups did not differ in terms of sex, age, BP, GFR, urine protein, CKD staging, glycemic levels, LDL and body composition. Also, no difference was found in baseline comorbid diseases, antihypertensive and hypoglycemic medications (Table 2).

**Table 1:** Baseline characteristics of patients.

Parameters	Pioglitazone 15 mg/day (N = 37)	Pioglitazone 7.5 mg/day (N = 38)	P value
Male (N, %)	17 (45.9%)	13 (34.2%)	0.300
Age (years)	61.8±11.6	63.9±12.8	0.460
Systolic blood pressure (mmHg)	135±19	133±19	0.260
Diastolic blood pressure (mmHg)	80±11	75±12	0.700
Glomerular filtration rate (mL/min/1.73 m^2^)	69.7±31.9	60.6±33.6	0.230
CKD stages (N, %)			0.340
Stage II	11 (29.7%)	12 (31.5%)	
Stage III	12 (32.4%)	16 (42.2%)	
Stage IV	10 (27.0%)	9 (23.6%)	
Urine protein creatinine ratio (mg/gCr)	1.1±2.6	0.6±1.1	0.220
Fasting plasma glucose (mg/dL)	199.5±87.2	174.8±77.4	0.200
HemoglobinA1C (%)	9.2±1.8	8.9±1.4	0.540
LDL (mg/dL)	116.9±46.8	107.5±35.4	0.210
AST (U/L)	24.3±15.8	24.9±13.3	0.900
ALT (U/L)	26.3±17.7	26.2±18.3	0.250
**Body compositions**			
Body weight (kg)	68.9±13.4	67.6±13.8	0.690
Body mass index (kg/m^2^)	27.3±5.1	26.3±3.9	0.400
Body fat mass (kg)	23.9±10.3	22.9±7.7	0.620
Total body water (L)	33.2±6.5	32.7±6.5	0.880
Extracellular water (L)	13.2±2.6	12.9±2.4	0.980

Data are number with percentage or mean ± SD and none of any parameter reached statistical significant p≥0.05

**Table 2:** Baseline comorbid diseases, antihypertensive and hypoglycemic medications.

Parameters	Pioglitazone 15 mg/day (N = 37)	Pioglitazone 7.5 mg/day (N = 38)
**Comorbid diseases**		
Hypertension (N, %)	34 (91.9%)	33 (86.8%)
Cerebrovascular disease (N, %)	2 (5.4%)	1 (2.6%)
Dyslipidemia (N, %)	34 (91.9%)	32 (84.2%)
Cardiovascular disease (N, %)	3 (8.1%)	7 (18.4%)
**Antihypertensive agents**		
Angiotensin converting enzyme inhibitor (N, %)	13 (35.1%)	6 (15.7%)
Angiotensin II receptor blocker (N, %)	8 (21.6%)	11 (28.9%)
Beta-blocker (N, %)	4 (10.8%)	10 (26.3%)
Calcium channel blocker (N, %)	18 (48.6%)	16 (42.1%)
Diuretic (N, %)	3 (8.1%)	3 (7.8%)
Direct vasodilator (N, %)	1 (2.7%)	0
**Hypoglycemic agents**		
Insulin (N, %)	17 (45.9%)	11 (28.9%)
Sulfonylurea (N, %)	20 (54.1%)	18 (47.3%)
Biguanide (N, %)	25 (67.6%)	17 (44.7%)
Alpha glucosidase inhibitor (N, %)	2 (5.4%)	2 (5.2%)

Data are number with percentage and none of any parameter reached statistical significant p≥0.05

### Glycemic and metabolic control during the study

**Fig 2:**
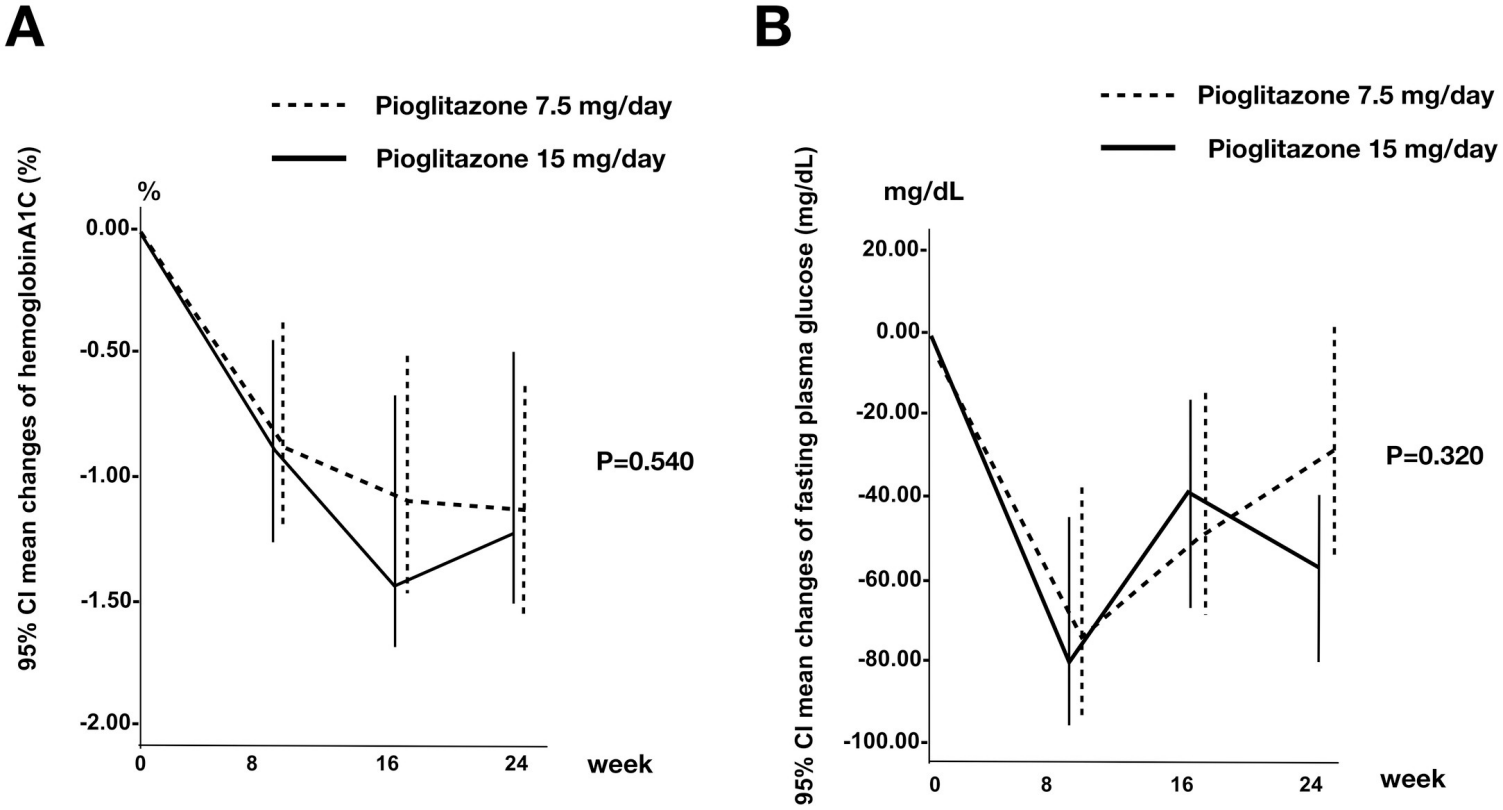
Mean changes in FFG (A) and HbA1C (B) from baseline during 24 weeks of treatment. Pioglitazone 7.5 mg is represented by a dotted line and pioglitazone 15 mg is represented by a black line. None of the intergroup differences reached statistical significance [mean change difference of FPG (-21.7 mg/dL, 95% CI -65.8 to 22.4) and HbA1C (0.3%, 95% CI -0.6 to 1.1)].

At the end of the 24 weeks, the mean FPG level significantly decreased from 174.8±77.4 to 157.3±43.2 mg/dL in the low dose pioglitazone group (p<0.05) and from 199.5±87.2 to 151.4±48.1 mg/dL in the standard dose pioglitazone group (p<0.05). Similarly, the mean HbA1C level significantly decreased from 8.9±1.4 to 7.6±0.9% in the low dose pioglitazone group (p<0.05) and from 9.2±1.8 to 7.9±1.4% in the standard dose pioglitazone group (p<0.05). No difference was observed between the low and standard dose pioglitazone groups regarding mean change difference of FPG (-21.7 mg/dL, 95% CI -65.8 to 22.4) and HbA1C (0.3%, 95% CI -0.6 to 1.1) during the study (Fig 2).

SBP, DBP, renal function, urine protein, urine sodium, serum AST, ALT and LDL did not significantly change in either group throughout the study (Table 3). Additionally, during the 24-week study period, no serious adverse events related or unrelated to pioglitazone were reported in both groups including drug-induced hepatotoxicity, severe hypoglycemia (blood glucose<70 mg/dL) and congestive heart failure.

**Table 3:** Changes in clinical and laboratory measurements.

Mean changes	Pioglitazone 15 mg/day (N = 37)	Pioglitazone 7.5 mg/day (N = 38)	Mean change difference between groups (95% CI)	P-value
Systolic blood pressure (mmHg)	0.3±17.3	1.5±20.1	-1.2 (-10.9, 8.4)	0.802
Diastolic blood pressure (mmHg)	-2.6±11.7	-0.3±9.9	-2.4 (-8.0, 3.3)	0.411
Glomerular filtration rate (mL/min/1.73 m^2^)	-3.8±8.1	0.1±9.5	-3.9 (-8.4, 0.7)	0.092
Urine protein creatinine ratio (mg/gCr)	-0.13±1.07	-0.01±0.28	-0.1 (-0.5, 0.3)	0.576
Urine sodium (mEq/g creatinine)	-0.01±1.5	0.2±0.8	-0.2 (-0.8, 0.4)	0.472
Serum LDL (mg/dL)	-6.6±40.2	5.7±32.6	-12.2 (-31.5, 7.1)	0.210
Serum AST (U/L)	-0.5±14.5	-0.1±10.3	-0.4 (-7.1, 6.3)	0.908
Serum ALT (U/L)	-4.2±15.1	0±12.7	-4.2 (-11.6, 3.2)	0.256

Data are number with percentage or mean ± SD and none of any parameter reached statistical significant p≥0.05.

### Edema status and body compositions

**Fig 3:**
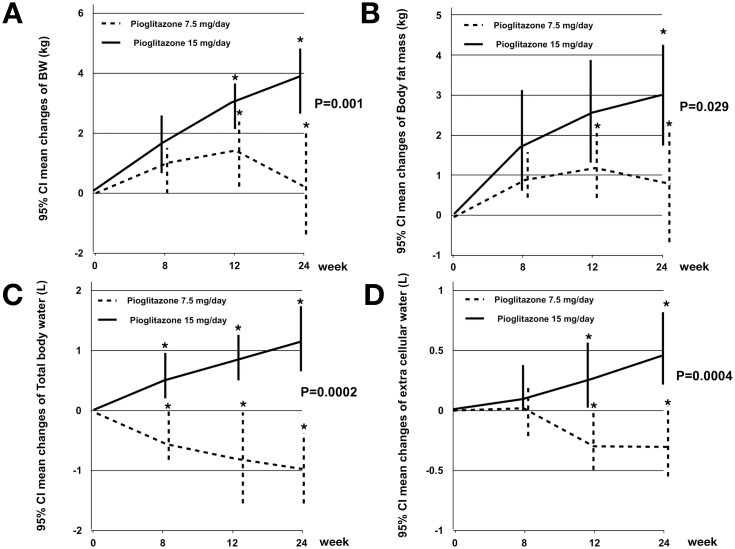
Mean changes in body weight (A), body fat mass (B), total body water (C) and extracellular water (D) from baseline during 24 weeks treatment. Pioglitazone 7.5 mg is represented by a dotted line and pioglitazone 15 mg is represented as a black line. Body weight (3.3 kg, 95% CI 1.3 to 5.2), body fat mass (2.2 kg, 95% CI 0.2 to 4.1), total body water (0.7 L, 95% CI 0.3 to 2.2) and extracellular water (0.4 L, 95% CI 0.1 to 1.1) increased significantly in the 15 mg pioglitazone group compared with the 7.5 mg pioglitazone group (P <0.05).

After 24 weeks of treatment, peripheral edema occurred at 17.0% in the standard dose pioglitazone group and 5.3% in the low dose pioglitazone group (P = 0.07). Compared with patients receiving 7.5-mg pioglitazone, patients receiving 15-mg pioglitazone had a significantly modest weight gain [3.5±3.2 vs. 0.2±4.4 kg with mean change difference between groups 3.3 kg (95% CI 1.3 to 5.2)]. The body composition measured by DSM-BIA method, total fat mass [2.9±3.7 vs. 0.7±3.6 kg with mean change difference between groups 2.2 kg (95% CI 0.2 to 4.1)], total body water (1.1±1.9 vs. -0.6±1.7 L with mean change difference between groups 0.7 L (95% CI 0.3 to 2.2)] and extracellular water [0.5±0.9 vs. -0.3±0.8 L with mean change difference between groups 0.4 L (95% CI 0.1 to 1.1)] in the 15-mg pioglitazone group was significantly greater than that in the 7.5-mg pioglitazone group (p<0.05) (Fig 3).

## Discussion

The present study constitutes the first randomized, controlled trial of oral 7.5 mg pioglitazone among patients with T2DM with CKD compared with pioglitazone 15 mg/day in terms of improving glycemic control and side effects. We did not observe any difference in the glucose control effects between the two groups, but 7.5-mg pioglitazone significantly decreased the risk of weight gain and increased fat mass, total body water and extracellular water. Taken together, these results serve as “proof of concept” that 7.5 mg pioglitazone improved TZDs side effects related to weight gain and fluid retention among patients with CKD.

TZDs has proven efficacy as hypoglycemic agents and being an insulin sensitizer, current clinical practice guideline recommendations state that TZDs, used among patients with CKD and TZDs, do not require dose adjustment. Regarding patients with renal impairment, risk of hypoglycemia does not increase [14, 15]. Though the guidelines approve treatment using TZDs among patients with CKD, the recommendations are mainly based on pharmacokinetics of TZDs. However, significant correlations between the dose of TZDs and weight gain were observed [16] and data from meta-analysis of randomized control trial among T2DM patients with renal impairment showed that TZDs significantly increased weight (mean difference 3.2 kg, 95% CI 2.3 to 4.2) and risk of edema (RR 2.9, 95% CI 1.2 to 7.2) [6]. Among Japanese women, 7.5-mg and 15-mg pioglitazone exhibited similar hypoglycemic effects, while a significantly higher percentage change of body weight and edema were associated with a higher dose [12]. Similarly, among Indian subjects, no difference was found between glycemic control in the 7.5-mg and 15-mg pioglitazone groups while significantly more weight gain was observed in the standard dose pioglitazone groups [17, 18]. Overall, this was consistent with our findings in that a considerably high percentage of peripheral edema, increased body weight, body fat and body fluid were observed among patients treated with 15-mg pioglitazone. However, a weight gain of 1 to 2 kg was found in long term TZDs treatment of T2DM patients. Whereas, our study showed high weight gains of 3.3 kg, it might be detected in the high risk setting of fluid retention and increased body fat including the combination of insulin therapy, being older, presenting T2DM with renal impairment and albuminuria. [19, 20]

Fluid retention and body weight gain with a 6 to 7% increase in blood volume among healthy volunteers induced by TZD treatment are caused by increased fluid reabsorption in the collecting duct and increased vascular permeability [10]. That increased plasma volume is related to TZDs has already been documented [21]. Clinical studies have shown that edema develops in approximately 3 to 5% of patients taking TZDs, with the incidence increasing to 10 to 15% among those receiving concurrent insulin therapy. A high susceptibility to the development of fluid retention has been attributed to T2DM subjects with advanced age, longstanding diabetes, insulin treatment, pre-existing edema, established cardiovascular disease and CKD [19, 20]. We also found that total body water and extracellular water increased significantly after treatment with 15-mg pioglitazone among patients with T2DM with CKD.

Moreover, weight gain from TZDs was associated with increased subcutaneous adipose tissue, decreased visceral fat and overall decreased ratio of visceral to subcutaneous fat [22, 23]. Among Caucasian patients with T2DM, a trend was observed for increased total body fat with TZDs (mean +1.02% body weight) and decreased total body fat in the placebo group (mean -0.54% body weight) [22]. Among Japanese women, the percentage change of body fat during the 24-week treatment in the 7.5 mg pioglitazone group was less than that in the 15-mg pioglitazone group (1.9 vs. 4.7%) [12]. The weight gain associated with TZDs treatment may be attributed to this change in fat distribution effect and total body fat. Overall, this was consistent with our findings in that significantly increased body fat was found among patients treated with 15-mg pioglitazone compared with 7.5 mg pioglitazone.

TZDs improves glycemic control through the dose-dependent enhancement of β-cell function and improved insulin sensitivity [24]. We did not detect any difference in the glucose control effects between the two groups, but responsiveness to pioglitazone was found to be relatively better in the 15-mg than in the 7.5-mg group in our study [mean changes in FPG -42.4±65.3 vs. -20.8±102.4 mg/dL, P = 0.32 and mean changes in HbA1c -1.4±1.5 vs. -1.1±1.6%, P = 0.54). However, the HbA1c reductions with an overall decrease of 1.1 to 1.4% in our study were higher than those in related studies with an overall decrease HbA1c between 0.4 and 0.7%. [11, 12, 18, 25] HbA1c changes were directly related to baseline HbA1c and our patients had relatively higher baseline HbA1c levels compared with related studies (HbA1c 9 vs 7–8%). Furthermore, recent meta-analysis among patients with CKD has suggested that no significant difference existed in the risk of hypoglycemia between TZDs and control groups. [6] However, hypoglycemia is a common adverse event in other hypoglycemic agents. [26] This was consistent with our findings suggesting no subjects presented hypoglycemia in our study.

Several limitations were associated with the present study. First, the long term outcomes and serious side effects especially heart failure from pioglitazone treatment among patients with T2DM could not be demonstrated in this study. No proof was evident that the increasing quantity of total body water and fat would have long term effects on clinical endpoints. Additional research is needed to confirm these results and determine long term clinical outcomes. Second, the study included its relatively small size of patients to assess differences in glycemic control, and visceral fat was not assessed. The strength of the study stemmed from the comprehensive body composition assessment of the patients’ responses to pioglitazone using the BIA method. Although DSM-BIA using the In-Body (720) body composition analyzer enhanced accuracy, body composition studies using the technique had reported mixed results, especially in the estimations of percentage fat mass.

## Conclusion

Our study demonstrates that pioglitazone 7.5 mg/day as an add-on to the standard therapy among Thai patients with T2DM and CKD is equally efficacious as the 15-mg doses of pioglitazone. The 7.5-mg pioglitazone therapy is more favorable for body weight, body fat and body water as compared with the 15-mg pioglitazone therapy. This may have a therapeutic role in modifying the dose of pioglitazone among patients with CKD with risk of fluid retention or weight gain.

## Supporting information

S1 File This file contains the detailed information about data, and parameters that were used to generate the results presented in this manuscript. (XLSX)

S1 Checklist (DOC)

S1 Protocol (DOCX)
