# Supplementary material for: Safety and efficacy of low dose pioglitazone compared with standard dose pioglitazone in type 2 diabetes with chronic kidney disease: A randomized controlled trial
Source: PLoS One. 2018 Oct 31;13(10):e0206722. doi: 10.1371/journal.pone.0206722 (PMC6209355; doi:10.1371/journal.pone.0206722)
Supplement: S1 Protocol — (DOCX) [file pone.0206722.s003.docx]

**Low Dose Pioglitazone Proposal**

| 1. ***Study title:*** *Safety and Efficacy of Low Dose Pioglitazone Compared with Standard Dose Pioglitazone in Type 2 Diabetes with Chronic Kidney Disease: a Randomized Controlled Trial* |
| --- |
|  |
| ***2. The purpose and rationale for the study*** |
| Throughout the world, chronic kidney disease (CKD) is a growing health concern because of its increasing prevalence and incidence rates and dismal outcomes. Despite technical advances in dialysis and transplantation, the prognosis of kidney failure remains poor. The U.S. Renal Data System provides statistics on individuals with end-stage renal disease (ESRD), which include individuals with kidney failure who are receiving dialysis, as well as kidney transplant recipients at all stages of CKD. This U.S. registry reported more than 76,500 deaths in patients with ESRD in 2001, an annual mortality rate in excess of 20% ^1^. By 2010, there will be a projected 160,000 new dialysis-dependent patients/year, double the current rate ^2^. The average life span of a patient entering a long-term dialysis program is 20-25 years less than that of the normal age-sex-race-matched U.S. population over the age of 45 ^3^. Among many factors, including treatment characteristics and co-morbid conditions, protein and calorie malnutrition has been shown to be a major risk factor for increased mortality in the ESRD patient population.^4^  Type 2 diabetes mellitus (T2DM) is one of the most important health problems and its disease prevalence has been increasing steadily all over the world. Similarly, the rising incidence of the chronic kidney disease (CKD) creates major problems for both healthcare systems and economies in future years ^5^. T2DM is often associated with CKD, and for 30 to 50% of patients receiving dialysis therapy, diabetes is the primary cause of end stage renal disease (ESRD)^6,7^. Identification and diagnosis of CKD is important to optimize clinical management recommendations for this complex patient population.  Therapeutic options for patients with T2DM and CKD are limited because the reduced glomerular filtration rate (GFR) results in an accumulation of certain drugs potentially leading to adverse side effects ^8^. Currently, thiazolidinediones (TZDs), synthetic exogenous agonists of the nuclear peroxisome proliferator-activated receptor-gamma (PPARγ), increase insulin sensitivity and inhibit adipose tissue lipolysis as well as possess anti-inflammatory and other beneficial vascular effects ^9^. TZDs undergo hepatic metabolism and has been demonstrated to be effective without increasing the risk of hypoglycemic episodes among patients with CKD ^10^. The pharmacokinetic profile of TZDS, is similar among subjects with normal or impaired renal function, remaining unaffected even by hemodialysis ^11^. Therefore, dosing of TZDs is not a required adjustment among patients with CKD.  Unfortunately, fluid retention and edema have emerged as the most common and serious side effects of TZDs and have become the most frequent cause of discontinued therapy ^12,13^. The mechanism through which TZDs induces fluid retention results from an increase in tubular sodium and water reabsorption in the collecting tubule and an increase in vascular permeability growth factors ^14^. Currently, using pioglitazone 15 to 45 mg daily is recommended for patients with T2DM. Patients with CKD might constitute a high risk population of TZDs related to fluid retention. Reducing fluid retention with low dose medications is important among those with CKD to continue the beneficial effects to optimize glycemic treatment without affecting the renin-angiotensin-aldosterone system and natriuretic peptides^15,16^. |
|  |
| ***3. The objective(s) and endpoint(s), if different from the objective(s)*** |
| - To evaluate the effects of low-dose pioglitazone (7.5 mg/day) on glucose metabolism compared with a standard-dose of pioglitazone (15.0 mg/day) among Thai patients with T2DM with CKD. - To determine side effects related to weight gain and fluid retention and the incidence of edema of low-dose pioglitazone (7.5 mg/day) compared with a standard-dose of pioglitazone (15.0 mg/day) among Thai patients with T2DM with CKD. |
|  |
| ***4. The study design*** |
| **A randomized, open-label controlled clinical trial.**  **Method**   1. The study will be started after the institute review board approval, Royal Army Medical Department. 2. Electronic case record form will be designed with subject code that personal subject’s data is not identified. 3. Patient screening will be conducted according to inclusion criteria and exclusion criteria. 4. Subjects will be divided into 2 groups (low and standard dose pioglitazone) by block randomization. The randomization and drug dispensing will be done by the investigator. The inform consent form will be read by the research staffs to the subjects and will be signed by every participated subjects. 5. The intervention group will receive pioglitazone 7.5 mg, 1 tablet 30 minutes before meal, once a day in the morning and the control group will receive the pioglitazone 15 mg as prior to the study. A recommendation card with detail of side effect and drug interaction will be provided to each subject who receives pioglitazone. 6. Five mL of blood will be collected along with 2 urine containers (at least 2 mL of urine per container) prior to the first treatment for the following items: CBC, fasting blood glucose, serum albumin, HbA1C, lipid profiles, BUN, creatinine, electrolyte, and liver function test. Body composition was assessed immediately after dialysis. Dual energy x-ray absorptiometry (DEXA) scan will be used to measure lean body mass and fat mass in grams using a whole-body scan. 7. At week 4, 8, 12, 16, 20, 24 visit, the investigator does the physical examination, symptom and drug adverse event interview, and laboratory testing such as CBC, fasting blood glucose, serum albumin, HbA1C, lipid profiles, BUN, creatinine, electrolyte, and liver function test. DEXA scan will be repeated. Contact can be made to the investigator for 24 hours regarding to any abnormal event occurs. |
|  |
| ***5. Treatment information, if applicable (e.g. for each study treatment, the dose, form, route, frequency, and duration, and if there is a dose-escalation, the dosing schema and rationale for the starting dose).*** |
| The intervention group will receive pioglitazone 7.5 mg, 1 tablet 30 minutes before meal, once a day in the morning and the control group will receive pioglitazone 15 mg as prior to the study. |
|  |
| ***6. Key milestones and estimated timelines.*** |
| **- STUDY PERIOD: 24 weeks/subject, approximately 1 year for data collection**  **-STUDY FINAL REPORT: January 2014, and December 2015 for study presentation and writing report** |
|  |
| ***7. The Investigator-Sponsor’s name, mailing address, phone number, and any other necessary contact information (e.g. email address, fax number, or both).*** |
| **Primary Investigator:** Khanin Watanakijthavonkul  **Affiliation:** Division of Nephrology, Department of Medicine, Phramongkutklao Hospital and College of Medicine  **Address:** 315 Rajavithi Road, Division of Nephrology, Department of Medicine, Phramongkutklao Hospital and College of Medicine, Bangkok, Thailand, 10400  **Phone:** 6626444676  **Fax:** 6626444676  **Email:** [asusa23@gmail.com](mailto:asusa23@gmail.com)  **Co-investigator(s): Bancha Satirapoj, M.D.**  **Affiliation:** Division of Nephrology, Department of Medicine, Phramongkutklao Hospital and College of Medicine  **Address:** 315 Rajavithi Road, Division of Nephrology, Department of Medicine, Phramongkutklao Hospital and College of Medicine, Bangkok, Thailand, 10400  **Phone:** 6626444676  **Fax:** 6626444676  **Email:** [satirapoj@yahoo.com](mailto:satirapoj@yahoo.com) |
|  |
| ***8. Any currently known location(s) where the study is to be conducted, including the name and full address of the institution(s) that are to be used in the study* *(including country)*** |
| Department of Medicine, Phramongkutklao hospital  315 Rajavithi Road, Phramongkutklao Hospital and College of Medicine, Bangkok, Thailand, 10400 |
|  |
| ***9. Methodology for data collection, including safety information*** |
| Electronic case record form will be designed with subject code that personal subject’s data is not identified. |
|  |
| ***10. Details of the proposed study population (including number of subjects, subject demographics [e.g. age, sex, and other key characteristics], and other key eligibility criteria).*** |
| **Expected number of subjects: 74 subjects**  **Inclusion criteria**   1. Age 18 years or older 2. Diagnosed T2DM with stable glycemic treatment with HbA1c>8% at least 12 weeks and diagnosed CKD according to [Kidney Disease: Improving Global Outcomes](https://www.google.co.th/url?sa=t&rct=j&q=&esrc=s&source=web&cd=8&cad=rja&uact=8&ved=0ahUKEwid7tCq8rPLAhVDBY4KHaXbAs8QFggyMAc&url=https%3A%2F%2Fwww.guideline.gov%2Fbrowse%2Fby-organization.aspx%3Forgid%3D2349&usg=AFQjCNFB0TRXT4TlgKTcrEguhdpN6NSZxg) (KDIGO) 2012 definition 3. No history of treatment with TZDs within 12 weeks before starting the study 4. Normal liver function, which referred to AST less than 105 U/L and ALT less than 120 U/L 5. Subject with signed consent form.   **Exclusion criteria**   1. Active malignancy 2. Severe heart, lung or liver disease, stroke, chronic infection, e.g., tuberculosis within one year of starting the study 3. Limited life expectancy within 12 months, edematous state from any cause 4. Specific contra-indications to pioglitazone including increased serum levels of liver enzyme (aspartate aminotransferase [AST] or alanine aminotransferase [ALT] >2.5 times the upper limit of normal), and history of bladder malignancy. 5. Subject without signed consent form   **Withdrawal or termination criteria**   1. The occurrence of heart failure or volume overload after drug administration which resulted in treatment needed or consent form withdrawal. 2. The occurrence of side effect which resulted in consent form withdrawal such as dizziness that affect driving, diarrhea that affect routine life. 3. The occurrence of severe infection, cardiac arrhythmia, myocardial infarction and cardiovascular hospitalization.   **Subject allocation**  Block randomization divides subjects into low dose group and standard dose group equally. A randomization in each group is conducted by dividing each subgroup into 20 groups with the setting number 0-19. The even number group, classified as A, is the intervention group. And the odd number group, classified as B, is the control group. |
|  |
| ***11. Key study assessments and procedures*** |
| The level of glycemic control is monitored by hemoglobinA1C and fasting plasma glucose.  Direct segmental multifrequency bioelectrical impedance analysis (DSM-BIA) is performed using the In-Body (720) body composition analyzer. This equipment has been shown to have high test-pretest reliability and accuracy.^17^ The spectrum of electrical frequencies is used to predict body composition, total fat mass, intracellular water (ICW) and extracellular water (ECW) compartments of the total body water (TBW) in the various body segments. |
|  |
| ***12. Statistical justification for the study design, including sample size calculations, and analysis plan.*** |
| The sample size was determined by using change in body fluid as the primary outcome measure and by extrapolating expected changes and standard deviation from data in the study of the effect of pioglitazone in T2DM patients. The target sample size was 37 subjects per group that will provide 80% power to detect a significant effect, using a 2-sided, *p*=0.05. **Formula:**  **Value description**  **$\alpha$ = 0.05 (two-tail) $Z_{0.025}$ = 1.96**  **$\beta$ = 0.20 $Z_{0.200}$ = 0.84**  **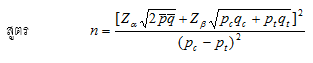**  **n = **  **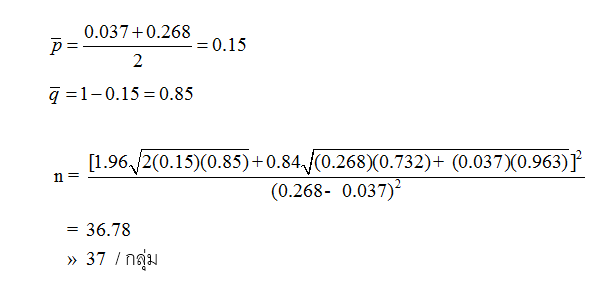**  By the calculation, the quantity of subject per group is 37. But with the follow up, the 10% loss follow up of the calculated number is considered. Therefore, the quantity of subject per group is 40, total is 80.  Statistical analyses were performed using the Statistical Package for Social Sciences (SPSS) software version 11.5. Intention-to-treat analysis was used by replacing the missing data with series mean for each group. Both descriptive and inferential statistics were determined. A bi-directional α-level of significance was set at *p*= 0.05 for all measures.   - Descriptive statisticsa was used for demographic results which are expressed as mean (± standard deviation; SD) and percentage unless otherwise indicated. - Chi-square tests were used for evaluating categorical variables. - Continuous variables between study and control groups were compared with unpaired *t-*tests. - Continuous variables between baseline and at the end of study for each group of patients were compared by using paired student *t-* tests. - Analysis of covariance (ANCOVA) was used for controlling covariate factors if baseline data are different between the patient groups. - One-way repeated analysis of variance (ANOVA) was used to examine the main effects of time when comparing data obtained at one time to data obtained at another time during the study period. - One-way repeated analysis of variance (ANOVA) with between-subjects factor was used to determine the main effects of overall difference between the control and the study groups with respect to the mean of the data. |
|  |
| ***13. Details of any clinical safety reporting processes and when applicable, specific risk management interventions to safeguard subjects*** |
| **Primary Investigator:** Khanin Watanakijthavonkul  **Affiliation:** Division of Nephrology, Department of Medicine, Phramongkutklao Hospital and College of Medicine  **Address:** 315 Rajavithi Road, Division of Nephrology, Department of Medicine, Phramongkutklao Hospital and College of Medicine, Bangkok, Thailand, 10400  **Phone:** 6626444676  **Fax:** 6626444676  **Email:** [asusa23@gmail.com](mailto:asusa23@gmail.com)  **Co-investigator(s): Bancha Satirapoj, M.D.**  **Affiliation:** Division of Nephrology, Department of Medicine, Phramongkutklao Hospital and College of Medicine  **Address:** 315 Rajavithi Road, Division of Nephrology, Department of Medicine, Phramongkutklao Hospital and College of Medicine, Bangkok, Thailand, 10400  **Phone:** 6626444676  **Fax:** 6626444676  **Email:** [satirapoj@yahoo.com](mailto:satirapoj@yahoo.com) |
|  |
| ***14. The public disclosure plan (including register postings, publications, communications, or presentations of the study results) and compliance of the Investigator-Sponsor’s public disclosure plan with the applicable controlled document(s): List Applicable SOPs here. This includes publications, communications, or presentations of the study results.*** |
| Plan to publish the study in Royal Thai Army Medical Journal and standard international medical journal. |

**References**

1. Bailie GR, Uhlig, K., and Levey, A. S. Clinical practice guidelines in nephrology: Evaluation, classification, and stratification of chronic kidney disease. Pharmacotherapy 2005;25:491-502.

2. Bailie GR. Dialysis outcomes quality initiative to kidney disease outcomes quality initiative: new clinical practice guidelines in nephrology -what the practicing pharmacist needs to know. Pharmacotherapy 2004;24:551-7.

3. Ikizler TA, et al. Association of morbidity with markers of nutrition and inflammation in chronic hemodialysis patients: a prospective study. Kidney Int 1999;55:1945-51.

4. Bergstrom J. Nutrition and mortality in hemodialysis. J Am Soc Nephrol 1995;6:1329-41.

5. Vejakama P, Ingsathit A, Attia J, Thakkinstian A. Epidemiological study of chronic kidney disease progression: a large-scale population-based cohort study. Medicine (Baltimore) 2015;94:e475.

6. Satirapoj B, Adler SG. Prevalence and Management of Diabetic Nephropathy in Western Countries. Kidney Dis (Basel) 2015;1:61-70.

7. Satirapoj B. Nephropathy in diabetes. Adv Exp Med Biol 2012;771:107-22.

8. Abe M, Okada K, Soma M. Antidiabetic agents in patients with chronic kidney disease and end-stage renal disease on dialysis: metabolism and clinical practice. Curr Drug Metab 2011;12:57-69.

9. Diamant M, Heine RJ. Thiazolidinediones in type 2 diabetes mellitus: current clinical evidence. Drugs 2003;63:1373-405.

10. Wang W, Zhou X, Kwong JSW, Li L, Li Y, Sun X. Efficacy and safety of thiazolidinediones in diabetes patients with renal impairment: a systematic review and meta-analysis. Sci Rep 2017;7:1717.

11. Budde K, Neumayer HH, Fritsche L, Sulowicz W, Stompor T, Eckland D. The pharmacokinetics of pioglitazone in patients with impaired renal function. Br J Clin Pharmacol 2003;55:368-74.

12. Arnouts P, Bolignano D, Nistor I, et al. Glucose-lowering drugs in patients with chronic kidney disease: a narrative review on pharmacokinetic properties. Nephrol Dial Transplant 2014;29:1284-300.

13. Nesto RW, Bell D, Bonow RO, et al. Thiazolidinedione use, fluid retention, and congestive heart failure: a consensus statement from the American Heart Association and American Diabetes Association. October 7, 2003. Circulation 2003;108:2941-8.

14. Yang T, Soodvilai S. Renal and vascular mechanisms of thiazolidinedione-induced fluid retention. PPAR Res 2008;2008:943614.

15. Kurisu S, Iwasaki T, Ishibashi K, et al. Effects of low-dose pioglitazone on glucose control, lipid profiles, renin-angiotensin-aldosterone system and natriuretic peptides in diabetic patients with coronary artery disease. J Renin Angiotensin Aldosterone Syst 2013;14:51-5.

16. Majima T, Komatsu Y, Doi K, et al. Safety and efficacy of low-dose pioglitazone (7.5 mg/day) vs. standard-dose pioglitazone (15 mg/day) in Japanese women with type 2 diabetes mellitus. Endocr J 2006;53:325-30.

17. Gibson AL, Holmes JC, Desautels RL, Edmonds LB, Nuudi L. Ability of new octapolar bioimpedance spectroscopy analyzers to predict 4-component-model percentage body fat in Hispanic, black, and white adults. Am J Clin Nutr 2008;87:332-8.
